# Disruption of Mitochondrial DNA Replication in *Drosophila* Increases Mitochondrial Fast Axonal Transport *In Vivo*

**DOI:** 10.1371/journal.pone.0007874

**Published:** 2009-11-17

**Authors:** Rehan M. Baqri, Brittany A. Turner, Mary B. Rheuben, Bradley D. Hammond, Laurie S. Kaguni, Kyle E. Miller

**Affiliations:** 1 Department of Zoology, Michigan State University, East Lansing, Michigan, United States of America; 2 Neuroscience Program, Michigan State University, East Lansing, Michigan, United States of America; 3 Center for Mitochondrial Science and Medicine, Michigan State University, East Lansing, Michigan, United States of America; 4 Department of Biochemistry and Molecular Biology, Michigan State University, East Lansing, Michigan, United States of America; 5 Department of Pathobiology and Diagnostic Investigation, Michigan State University, East Lansing, Michigan, United States of America; Brigham and Women's Hospital/Harvard Medical School, United States of America

## Abstract

Mutations in mitochondrial DNA polymerase (pol γ) cause several progressive human diseases including Parkinson's disease, Alper's syndrome, and progressive external ophthalmoplegia. At the cellular level, disruption of pol γ leads to depletion of mtDNA, disrupts the mitochondrial respiratory chain, and increases susceptibility to oxidative stress. Although recent studies have intensified focus on the role of mtDNA in neuronal diseases, the changes that take place in mitochondrial biogenesis and mitochondrial axonal transport when mtDNA replication is disrupted are unknown. Using high-speed confocal microscopy, electron microscopy and biochemical approaches, we report that mutations in pol γ deplete mtDNA levels and lead to an increase in mitochondrial density in *Drosophila* proximal nerves and muscles, without a noticeable increase in mitochondrial fragmentation. Furthermore, there is a rise in flux of bidirectional mitochondrial axonal transport, albeit with slower kinesin-based anterograde transport. In contrast, flux of synaptic vesicle precursors was modestly decreased in pol γ−α mutants. Our data indicate that disruption of mtDNA replication does not hinder mitochondrial biogenesis, increases mitochondrial axonal transport, and raises the question of whether high levels of circulating mtDNA-deficient mitochondria are beneficial or deleterious in mtDNA diseases.

## Introduction

Mitochondrial DNA (mtDNA) depletion, deletion and/or point mutations are implicated in many diseases. Mutations in DNA polymerase gamma (pol γ), the mtDNA replicase [1], cause several progressive diseases including Parkinson's disease [2], [3], Alpers' syndrome [4], and progressive external ophthalmoplegia [5]. Pol γ is the sole DNA polymerase responsible for mtDNA replication in animals. It is a highly accurate and processive protein complex comprising a 125 kD catalytic subunit (pol γ−α) and a 35 kD accessory subunit (pol γ−β) in *Drosophila*, encoded by the genes *tamas* and *pol γ−β*, respectively. Both subunits have been cloned and characterized extensively in *Drosophila* [6], [7] and in numerous other systems including humans [1]. At a cellular level, alteration of pol γ expression leads to depletion of mtDNA, disrupts the mitochondrial respiratory chain, and increases susceptibility to oxidative stress [8]–[12].

Mitochondria are distributed in neurons through a dynamic combination of transport and stopping (docking) events [13]. Fast axonal transport of mitochondria is carried out by molecular motors, conventional kinesin and cytoplasmic dynein, that utilize ATP to perform their function [14]–[17]. Healthy mitochondria have a high mitochondrial membrane potential that provides the electrochemical energy to drive ATP synthesis through oxidative phosphorylation [18]. Accordingly, the role of mitochondrial ATP production in regulating fast axonal transport has been the subject of several studies. Early *in vitro* studies designed to disrupt oxidative phosphorylation have suggested that it is essential for the maintenance of axonal transport [19]. However, other studies have presented conflicting results. For example, uncoupling agents CCCP and FCCP block all cytoplasmic transport while another uncoupler DNP has no effect [20]; complex III inhibitor antimycin increases retrograde transport with little effect on anterograde transport [13], as does the complex I inhibitor annonacin [21]. As the disruption of axonal trafficking is implicated in neuronal degeneration and is observed in diseases like Alzheimer's disease, Huntington's disease, spinobulbar muscular atrophy, Charcot-Marie-Tooth disease, *etc.* [22]–[26], the influence of genetic impairment of oxidative phosphorylation on axonal transport is especially relevant. We hypothesized that mutations in the accessory and catalytic subunits of pol γ would disrupt fast axonal transport. To investigate the influence of depleted mtDNA content on axonal trafficking and mitochondrial biogenesis, we studied transport dynamics in pol γ mutants of *Drosophila*.

## Results

### mtDNA Is Depleted in Mutants of pol γ

**Figure 1:**
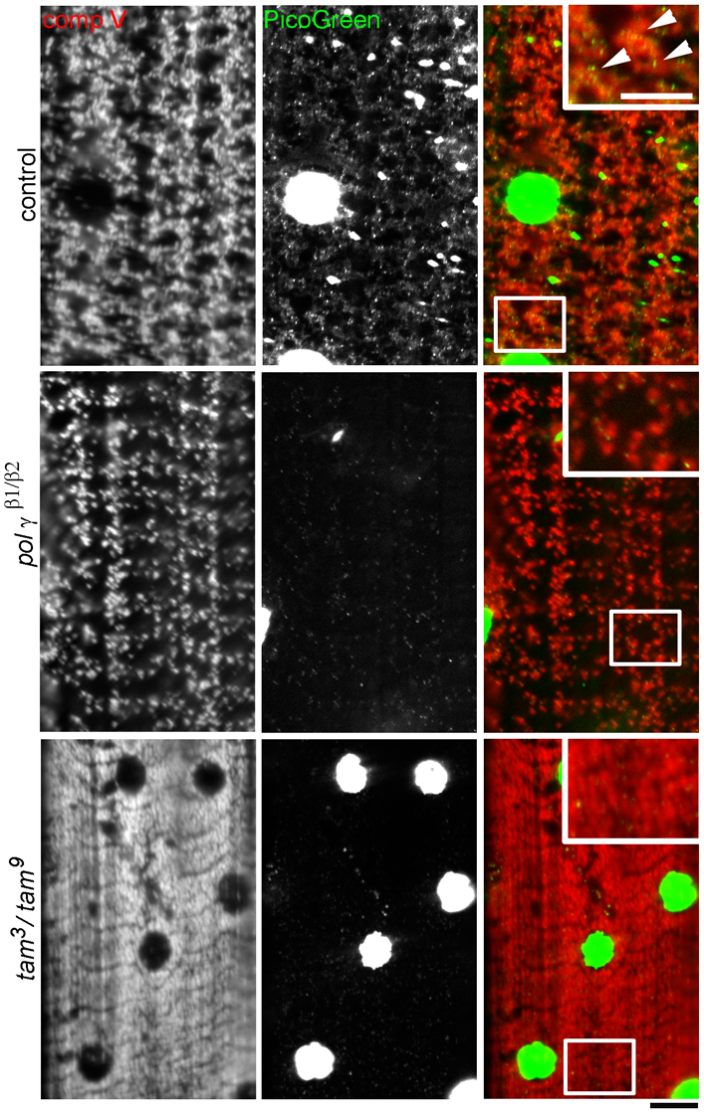
Mutations in the catalytic and accessory subunits of DNA polymerase γ impair mtDNA replication and decrease mtDNA content. An antibody against mitochondrial complex V (red) and the dye PicoGreen (green) are used to label mitochondria and mtDNA respectively, in muscles of wildtype control, *pol γ ^β1/β2^* and *tam* ^3^/*tam* ^9^ crawling 3^rd^ instar *Drosophila* larvae. Regular mitochondrial distribution is disrupted in *pol γ ^β1/β2^* and *tam* ^3^/*tam* ^9^ mutants and number of mtDNA nucleoids are significantly reduced. PicoGreen also labels the dsDNA of muscle nuclei that serves as internal control for the staining. Muscle nuclei appear smaller in the pol γ mutants. Because of the relatively high concentration of dsDNA in muscle nuclei, they appear saturated at offset levels required to visualize the smaller mtDNA nucleoids. Insets in the RGB merge show digitally magnified regions from the boxes and arrowheads indicate presence of mtDNA in control muscles and are absent in muscles of pol γ mutants. *tam* ^3^/*tam* ^9^ larvae have visibly higher mitochondrial density. Scale bars equal 10 µm.

**Figure 2:**
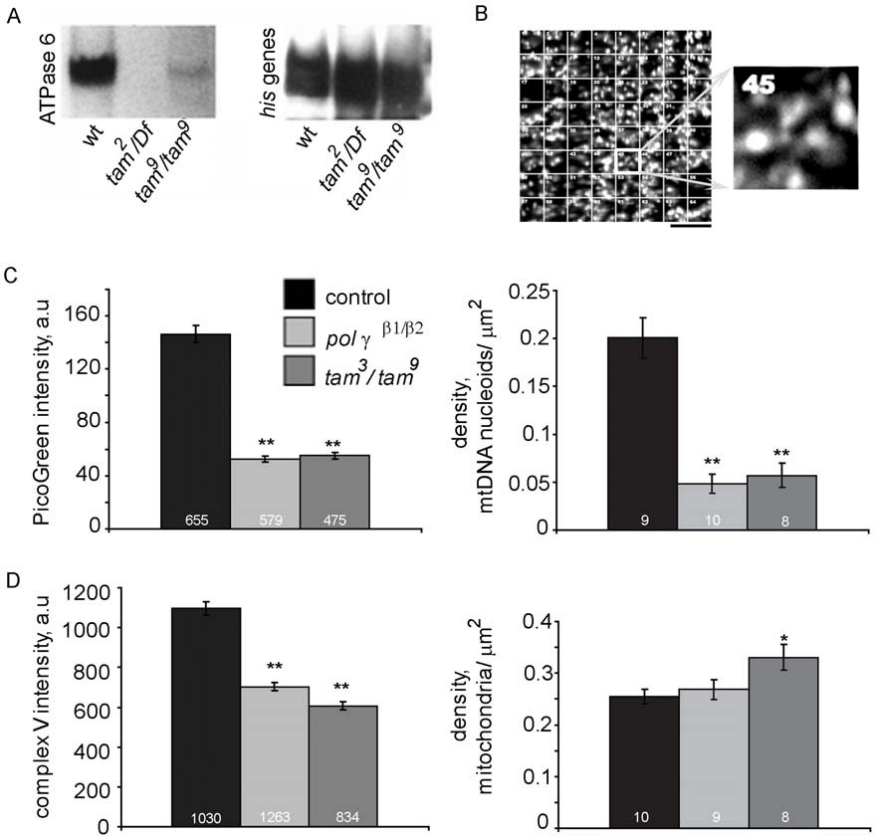
Mitochondrial density is higher in *tam^3^*/*tam* ^9^ mutants whereas density of mtDNA nucleoids is reduced. (A) Southern blot analysis of total DNA extract, hybridized with ^32^P-labeled DNA probes from the ATPase 6 gene of mtDNA shows a decrease in pol γ mutants compared to wildtype controls. A histone gene cluster (*his* genes) is used as a nuclear DNA control. (B) Density of mitochondria and mtDNA nucleoids is measured by dividing a 512×512 pixel frame of the muscle into a grid of 64 regions of interest (ROIs). A random list of numbers (1–64) was generated and the number of mitochondria/mtDNA nucleoids was counted in those ROIs for each frame. (C) Intensity of PicoGreen stained mtDNA (in arbitrary units, a.u); as well as density of mtDNA nucleoids was reduced significantly in pol γ mutant muscles. Numbers at the base of columns represent number of mtDNA nucleoids sampled and number of animals used, respectively. (D) Intensity of complex V stained mitochondria is reduced significantly in both pol γ mutants (in arbitrary units, a.u); while average mitochondrial density is increased moderately in *tam* ^3^/*tam* ^9^ mutant muscles. Numbers at the base of columns represent number of mitochondria sampled and number of animals used, respectively. Error bars represent 95% confidence intervals. * indicates p<0.05 and ** indicates p<0.001 from Student's t-test.

We disrupted mtDNA replication in *Drosophila* using mutations in the two subunits of pol γ, *pol γ-^β1/β2^* (accessory subunit mutant alleles) and *tam* ^3^/*tam* ^9^ (catalytic subunit mutant alleles) [9], [27]. To verify that mtDNA content is decreased in these backgrounds, we stained muscles of crawling third instar *Drosophila* larvae with the fluorescent dye PicoGreen (Figure 1). PicoGreen reliably labels mtDNA and has been used to detect mtDNA depletion in living human cells and rat liver mitochondria [28]. Because muscles are large, flat, and contain multiple nuclei that serve as internal controls for dsDNA, they are excellent for quantitative assessment of mtDNA depletion. We measured mtDNA depletion in muscles 6/7 in abdominal segments A3–A6. Muscle nuclei were stained brightly and displayed similar pixel intensity in wildtype, *pol γ-^β1/β2^* and *tam* ^3^/*tam* ^9^ mutants, suggesting that PicoGreen had been incorporated equally and sufficiently into dsDNA. Numerous mtDNA nucleoids were visible in the wildtype muscles, but were nearly absent at identical exposure levels in *pol γ-^β1/β2^* and *tam* ^3^/*tam* ^9^ mutants (Figure 1). The normal mitochondrial distribution and patterning in wildtype muscles is seen by co-immunostaining with an antibody against mitochondrial complex V. This patterning was severely disrupted in *pol γ-^β1/β2^* and *tam* ^3^/*tam* ^9^ mutants. In particular, some *tam* ^3^/*tam* ^9^mutants had visibly higher mitochondrial density and mitochondria appeared to be tightly packed instead of arranged in normal banded patterns. Quantitative analysis of PicoGreen staining in muscles reveals a significant decrease in density of mtDNA nucleoids in *pol γ-^β1/β2^* and *tam* ^3^/*tam* ^9^ mutants as compared to controls (Figure 2C). Further, the fluorescent intensity of PicoGreen stain is also reduced significantly (Figure 2C), suggesting lower mtDNA content in the existing nucleoids. This is the first study to visualize directly mtDNA depletion in muscles using tissue staining.

In order to validate these results using a molecular analysis, we evaluated mtDNA content and integrity in the pol γ mutants by quantitative Southern blotting. Total DNA from wildtype and mutant pol γ larvae late in the third instar was isolated and digested with *Xho*I, which cleaves *Drosophila* mtDNA once. A mtDNA probe encoding the ATPase 6 gene was used to determine mtDNA copy number and integrity, and a multiple-copy genomic probe of the nuclear histone gene cluster was used as a DNA loading control. Multiple analyses of mtDNA content demonstrated severe mtDNA depletion in pol γ mutants; mtDNA was nearly undetectable in *pol γ-α* mutant larvae (Figure 2A). Based on prior work that estimates that 2–10 molecules of mtDNA are found in each mitochondrion [28], [29], we make the conservative estimate that more than 90% of the mitochondria in *pol γ-^β1/β2^* and *tam* ^3^/*tam* ^9^ mutant backgrounds lack mtDNA.

### Lysosomal dsDNA Clusters Are Absent in pol γ-^β1/β2^ and tam^3^/tam^9^ Mutants

**Figure 3:**
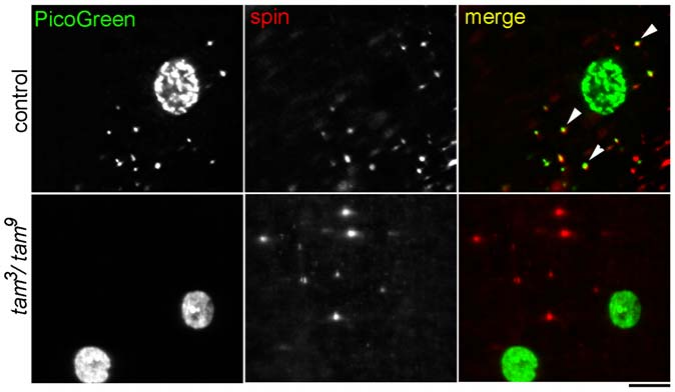
Lysosomal clusters of double-stranded DNA are absent in muscles of pol γ mutant larvae. Immunolabeling of late endosomal/lysosomal compartments with anti-spin antibody (red) reveals the presence of extra-nuclear dsDNA clusters (green) in these compartments in control muscle cells (arrowheads), presumably from mitochondria undergoing mitophagy. These dsDNA clusters are absent in the lysosomes of *tam* ^3^/*tam* ^9^ mutant muscles. Scale bar equals 10 µm.

Interestingly, control muscles regularly displayed numerous dense extra-nuclear clusters of dsDNA that did not colocalize with mitochondria, and these clusters were absent or rarely seen in *pol γ-^β1/β2^* and *tam* ^3^/*tam* ^9^ mutants (Figure 3). We investigated further the nature of these clusters by co-immunostaining with several probes and antibodies and found them to colocalize completely with anti-spin antibody (Figure 3, arrowheads). *Spin (Spinster)* encodes a multipass transmembrane protein that is localized to a late endosomal/lysosomal compartment [30]. Expectedly, not all anti-spin compartments have dsDNA but all dsDNA clusters colocalize with anti-spin, indicating that the extra-nuclear dsDNA clusters were always present inside lysosomes, whereas all lysosomes do not contain dsDNA clusters.

### Mitochondrial Density Is Higher in Muscles of tam^3^/tam^9^ Mutants

Mitochondrial density increases in the liver when pol γ is disrupted in humans [31]. Mitochondrial mass is also increased when mitochondrial transcription factor B2 is downregulated in *Drosophila* [32]. To determine whether mutations in pol γ altered mitochondrial density in *Drosophila*, we studied the spatial distribution of mitochondria in muscles 6/7 in abdominal segments A3–A6 of *tam* ^3^/*tam* ^9^ and *pol γ-^β1/β2^* larvae. A single confocal plane from immunolabeled images was cropped into 512×512 pixel frames and divided into a 64-square grid. A random list of squares was generated for sampling; the mitochondria in each square were counted and divided by the area to yield density in units of mitochondria/µm^2^ (Figure 2B). Average mitochondrial density registered a slight increase in *pol γ-^β1/β2^* mutants and was significantly higher in *tam* ^3^/*tam* ^9^ mutants (Figure 2D). Further, quantitative analysis of the average fluorescent intensity of anti-complex V staining is significantly reduced in *pol γ-^β1/β2^* and *tam* ^3^/*tam* ^9^ mutants (Figure 2D). Mitochondrial complex V, also called ATP synthase, is responsible for ATP synthesis. Reduction in anti-complex V staining suggests that in *pol γ-^β1/β2^* and *tam* ^3^/*tam* ^9^ mutants the overall health and ATP generation capacity of mitochondria is compromised. Together, our data demonstrate that the average mitochondrial density in muscles is not decreased in both mutants of mtDNA replication, and in fact may increase in *tam* ^3^/*tam* ^9^ mutants.

### Mitochondrial Density Is Higher in the Proximal Nerves of tam^3^/tam^9^ Mutants

**Figure 4:**
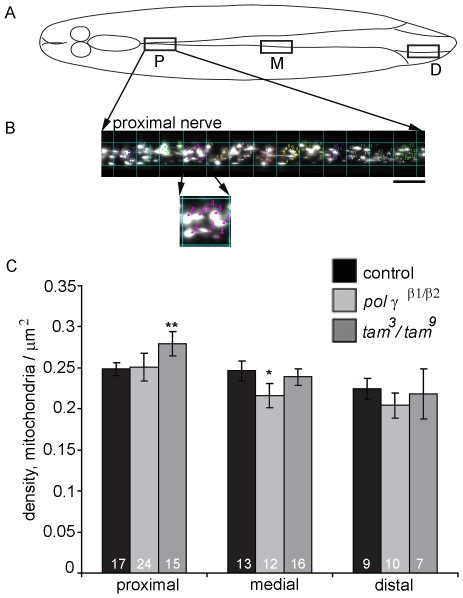
Mitochondrial density is increased in the proximal nerves of *tam^3^*/*tam* ^9^ mutant larvae. (A) Schematic illustration of the neuronal organization of the 3^rd^ instar *Drosophila* larva. The region in box P represents the area imaged for proximal, M for medial and D for distal nerve analysis. (B) The nerve was divided into 100 µm^2^ bins along its length and individual mitochondria counted in each bin and averaged over the area to yield mitochondrial density/µm^2^. Scale bar equals 10 µm. (C) Mitochondrial density is significantly increased in proximal region of nerves of *tam^3^*/*tam* ^9^ mutant larvae compared to wildtype controls. Density along the medial nerve and distal nerve remains unchanged in *tam^3^/tam^9^* mutants. Error bars represent 95% confidence intervals. * indicates p<0.05 and ** indicates p<0.001 from Student's t-test.

To evaluate the effect of mtDNA depletion on mitochondrial distribution in neurons, we measured mitochondrial density in the segmental nerves of flies with the genotypes *pol γ-^β1/β2^*; *UAS-mtGFP, D42-Gal4/+* and *tam* ^3^/*tam* ^9^; *UAS-mtGFP, D42-Gal4/+*. *UAS-mtGFP* encodes the S65T spectral variant of GFP fused at the N-terminus with the 31 amino acid mitochondrial import sequence from human cytochrome C oxidase subunit VIII. The *D42-gal4* driver predominantly expresses in motor neurons along with a few body wall sensory neurons, and the salivary glands in the larva [33]. As the cell bodies of motor neurons are located in the ventral nerve cord, regions of the segmental nerve that are close to the ventral nerve cord correspond to the proximal axonal regions of the motor neurons. 3-D reconstructed images from the proximal, medial and distal nerve region of *UAS-mtGFP; D42-gal4* expressing animals were used for the analysis (Figure 4A and B). Quantification of average mitochondrial density reveals a significant increase in the proximal segmental nerves of *tam* ^3^/*tam* ^9^ mutants while proximal nerves of *pol γ-^β1/β2^* mutants have mitochondrial densities similar to control. The medial and distal nerves in the same animals did not show any difference from control; in fact, *pol γ-^β1/β2^* mutants registered a slight reduction in mitochondrial density in the medial and distal regions (Figure 4C).

### Mitochondrial Ultrastructure Is Preserved in pol γ-^β1/β2^ and tam^3^/tam^9^ Mutants

Mitochondrial fragmentation has been characterized by an increase in numbers of small round mitochondria and by mitochondria with abnormal cristae [34]. To determine if mitochondria were fragmented or otherwise abnormal, muscle fibers, the segmental and intersegmental nerves, the small nerve branches within the body wall muscles, and neuromuscular junctions were examined using transmission electron microscopy. Third instar larvae prior to the wall climbing stage were selected for study to avoid any age-related degeneration that might occur in association with metamorphosis.

**Figure 5:**
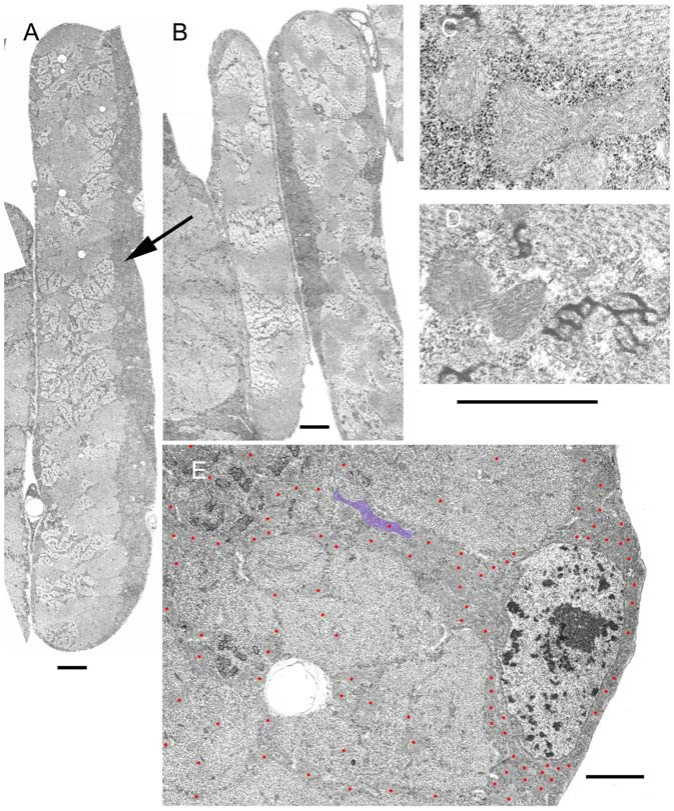
Mitochondrial morphology and distribution in larval muscles. (A) (muscle fiber 7) and (B) (muscle fibers 15 and 28) are montages of low power electron micrographs to illustrate whole muscle cells. (A and C) are from Canton S, and (B, D, and E) from *tam* ^3^/*tam* ^9^. In (A) and (B) the dark bands (arrow) along the edges of fibers consist of mitochondria and glycogen granules. The thickness of this layer can be seen to vary along the same fiber and between adjacent fibers particularly on (B). Mitochondria and glycogen are also found in streaks radiating inward from the edges of the fibers, largely in the vicinity of the Z bands (C, D, and E); their distribution gives rise to the banded patterns seen with immunolabeling (Figure 1). (E) Illustrates the increase in clusters of mitochondria around nuclei; the individual mitochondria have been indicated with a red dot at high magnification so their distribution can be visualized. The shape of a single mitochondrion was overlaid with blue to indicate relative size. No qualitative changes were detected between the structures of control (C) and *tam^3^/tam^9^* mutant mitochondria (D). Scale bars equal 2 µm for (A and B), 1 µm for (C, D, and E).

Mitochondria in *Drosophila* muscle are typically arranged in large masses along with glycogen granules between the sarcolemma and the columns of myofibrils. These groups of mitochondria are distributed irregularly around the circumference and along the length of the fiber, so that they may or may not be present in any given plane of section. There may be a thick layer on one side of the fiber and not on the other (Figure 5A, arrow). Mitochondria and glycogen also surround each muscle fiber nucleus and each neuromuscular junction. Smaller (cross-sectional diameter) mitochondria are found along the sarcoplasmic reticulum among the columns of myofilaments at the level of z-bands. No qualitative differences were detected in this general pattern of distribution among Canton-S and *D42*-Gal4 controls or among *pol γ-^β1/β2^* and *tam* ^3^/*tam* ^9^ mutants. The appearance of individual mitochondria also did not differ between mutants and controls. We found no examples either of mitochondria with crystalline formations or with striking abnormalities in the cristae (Figure 5), features that are typically associated with mitochondrial fragmentation [34]. Due to the small sample size, we did not conduct a statistical analysis of muscle fiber mitochondrial density.

**Figure 6:**
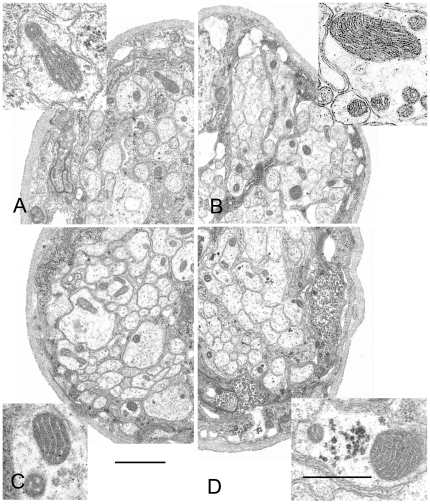
Mitochondrial ultrastructure in segmental nerves. Each quadrant shows a portion of a segmental nerve from each of the four types of animals that were examined. No differences in the nerve structure were noticed between control and mutant larvae. (A) Background control (*UAS-mtGFP; D42*-Gal4 in wildtype background); (B) Canton S; (C) *tam* ^3^/*tam* ^9^; and (D) *pol γ-^β1/β2^*. Cross-sections of mitochondria (insets) are found in about 1 out of every 5 axonal profiles in controls. The relative number of mitochondrial profiles in axons appeared to be slightly increased in *pol γ-^β1/β2^* and *tam* ^3^/*tam* ^9^ mutants, to around 1 out of 3 in a sample of 3 nerves each. Oblique or longitudinal sections show that axonal mitochondria can be quite long and threadlike, and range in diameter from 100 to 800 nm. Both large and small diameter mitochondria may be found side by side in the same axon (B, C, D). Glycogen granules are seen in some of the large diameter axons (D). Scale bars equal 1 µm for nerve quadrants and 0.5 µm for insets.

In nerves, the mitochondria in the axons and glia are smaller in diameter than those in muscle fibers. Typically only one profile of a mitochondrion is present in any given cross-section of a particular axon although exceptions are seen in which 5 or more are present in both control and mutant animals. Several to a dozen microtubules (depending on axon diameter) are also present, and occasionally clusters of glycogen granules are seen within axons or in glia. No qualitative differences in the structure of axonal or glial mitochondria were detected between the control and the mutant animals and cristae appeared normal (Figure 6). In neuromuscular junctions a mitochondrion may or may not appear in any given cross section; no difference in morphology in these mitochondria was seen between mutant and control animals. Together, these morphological observations suggest that mitochondria remaining in nerves and muscles at this stage are structurally normal. The similar range of sizes observed in the sampled populations together with the fluorescence observations suggest that mitochondrial fragmentation is not occurring in pol γ mutants, but modest increases in fission cannot be ruled out.

### Mitochondrial Flux Increases when mtDNA Replication Is Impaired

**Figure 7:**
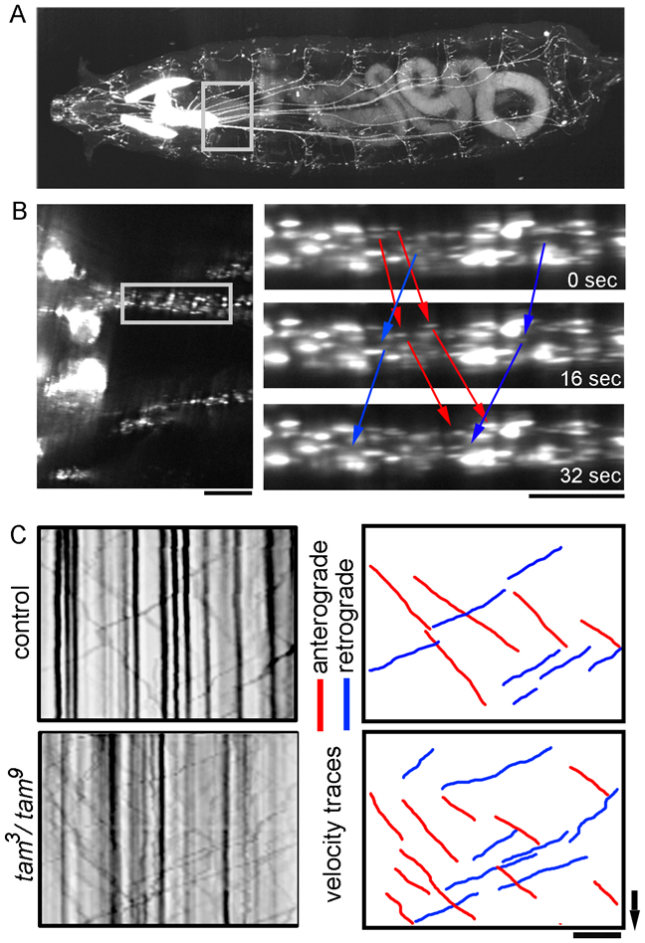
Measurement of flux and velocity of axonal transport from *Drosophila* segmental nerves. (A) 3-D reconstruction of a 3^rd^ instar *Drosophila* larva expressing *UAS-mtGFP* in segmental nerves under the *D42*-GAL4 driver. Mitochondria can be visualized in the CNS, salivary glands and the entire length of segmental nerves. There is some auto-fluorescence in the gut. A single optical plane from the proximal region of the two medial nerves was used for time lapse imaging as described in the methods section. (B) Kymographs were generated as described in the methods section. Dark vertical lines denote docked mitochondria; hand traced red lines depict mitochondria moving in the anterograde direction while blue lines depict retrograde movement. The slope of these lines yields velocity of transport. Arrow represents 20 s and the scale bar equals 10 µm.

**Figure 8:**
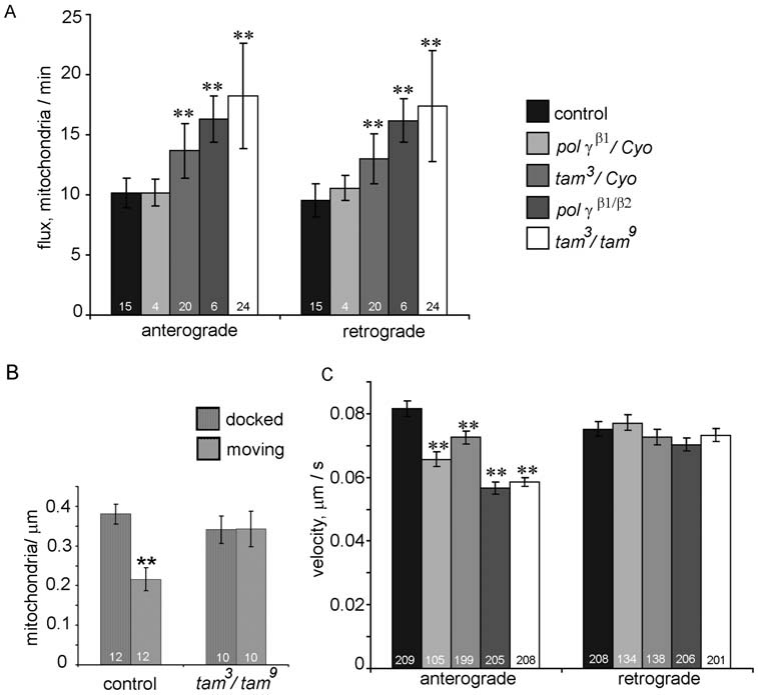
Mutations in pol γ increase mitochondrial flux in both directions but decrease only anterograde velocity. (A) Bidirectional mitochondrial flux is increased significantly in segmental nerves of heterozygous *tam^3^* and heteroallelic combination of *pol γ ^β1/β2^* and *tam* ^3^/*tam* ^9^; numbers at the base of columns indicate number of nerves assayed. (B) There is no significant change in the density of docked mitochondria in *tam* ^3^/*tam* ^9^ mutants; numbers at the base of columns indicate number of nerves assayed. (C) Average velocity of anterograde run is decreased significantly in heterozygous and heteroallelic pol γ mutants while average velocity of retrograde runs is affected; numbers at the base of columns indicate number of mitochondria traced. All error bars represent 95% confidence intervals. * indicates p<0.05 and ** indicates p<0.001 from Student's t-test.

To determine if neuronal mitochondrial transport was altered *in vivo*, we used the same methodology we developed for observing the transport of synaptic vesicle precursors in *Drosophila* [35]. In brief, intact third instar larvae were anesthetized and mounted between a slide and coverslip and time lapse images of GFP-labeled mitochondria were acquired (Figure 7A and B; see Materials and methods). The mitochondrial label (*UAS-mtGFP*) was expressed in motor neurons using the *D42-Gal4* driver. In control and mutant backgrounds, the number of GFP-labeled mitochondria that crossed a specific point across the nerve were counted, and the total was then divided by the ‘time of observation’ to yield ‘flux’ in terms of mitochondria/min. To measure these dynamics we generated kymographs, which are graphical records of transport with distance on the X-axis and time on the Y-axis (Figure 7C). Mitochondrial flux registered a striking increase in both directions in *pol γ-^β1/β2^* and *tam* ^3^/*tam* ^9^ mutants as compared to control animals (Figure 8A, Videos S1 and S2). Consistent with the varying severity of *pol γ-^β1/β2^* and *tam* ^3^/*tam* ^9^ mutant phenotypes, heterozygous *pol γ−^β1^/Cyo* does not show any significant difference from control, whereas a single copy of the mutation in the heterozygous *tam^3^/Cyo* animals is sufficient for a significant increase in bidirectional flux. The average flux values with standard deviations are summarized in Table 1. These data provide the first direct evidence that bidirectional mitochondrial transport increases when mitochondrial DNA replication is blocked.

**Table 1:** Axonal transport flux and velocity of mitochondria and synaptic vesicle precursors in 3^rd^ instar *Drosophila* larvae.

**Axonal Transport of Mitochondria**					
**FLUX (mitochondria/min)**	control	*pol γ-^β1^/Cyo*	*tam* ^3^/*Cyo*	*pol γ-^β1/β2^*	*tam* ^3^/*tam* ^9^
Anterograde	10.15±2.48	10.17±2.33	13.66±2.56	16.31±5.47	18.20±4.87
Retrograde	9.52±2.73	10.56±2.10	13.00±2.40	16.18±5.79	17.38±4.49
**VELOCITY (µm/s)**	control	*pol γ-^β1^/Cyo*	*tam* ^3^/*Cyo*	*pol γ-^β1/β2^*	*tam* ^3^/*tam* ^9^
Anterograde	0.08±0.01	0.06±0.01	0.07±0.01	0.05±0.01	0.05±0.01
Retrograde	0.07±0.01	0.07+0.01	0.07+0.01	0.07+0.01	0.07+0.01
**Axonal Transport of Synaptic Vesicle Precursors**					
**FLUX (syn. vesicles/min)**	control			*pol γ-^β1/β2^*	*tam* ^3^/*tam* ^9^
Anterograde	39.07±6.36			37.81±6.25	34.59±4.53
Retrograde	35.46±5.45			32.87±6.10	30.48±6.32
**VELOCITY (µm/s)**	control			*pol γ-^β1/β2^*	*tam* ^3^/*tam* ^9^
Anterograde	0.08±0.02			0.08±0.02	0.08±0.01
Retrograde	0.06±0.01			0.06+0.01	0.06+0.01

Axonal transport of mitochondria and synaptic vesicle precursors.

Flux and velocity values for bidirectional transport of mitochondria (*UAS mtGFP*) and synaptic vesicle precursors (*UAS-n-sybGFP*) in Drosophila larvae. Errors indicate standard deviation.

Next, we measured the propensity of mitochondria to remain stationary or remain motile in the same nerve regions used to measure flux. We found that there was no significant change in the number of docked mitochondria in control and *tam* ^3^/*tam* ^9^ mutants (Figure 8B). The ratio of docked versus moving mitochondria increases in *tam* ^3^/*tam* ^9^ mutants because of higher number of motile mitochondria.

### Velocity of Anterograde Mitochondrial Transport Is Reduced whereas Retrograde Velocity Is Unchanged

To determine if depletion of mtDNA altered the more subtle aspects of transport, we measured the velocity of mitochondrial transport in *pol γ-^β1/β2^* and *tam* ^3^/*tam* ^9^ mutants, as well as heterozygous flies with single copy of the mutations. In all conditions, we found that kinesin-based anterograde velocity was reduced significantly whereas dynein-based retrograde velocity was maintained at the same rate as control animals (Figure 8C). The average velocity values with standard deviations are summarized in Table 1. Although this impairment in axonal transport is minimal, it could contribute to peripheral neuropathy over a sustained time period as observed with some POLG1 mutations [36], [37]. These results raise the question if decreased anterograde velocity is due to a global disruption of axonal transport such as an alteration in ATP or ADP levels [19], [38], the formation of ‘clogs’ or blocks along the axon [39], or disruption of the integrity of microtubules [40].

### Synaptic Vesicle Precursor Transport Is Largely Unaffected in pol γ-^β1/β2^ and tam^3^/tam^9^ Mutants

**Figure 9:**
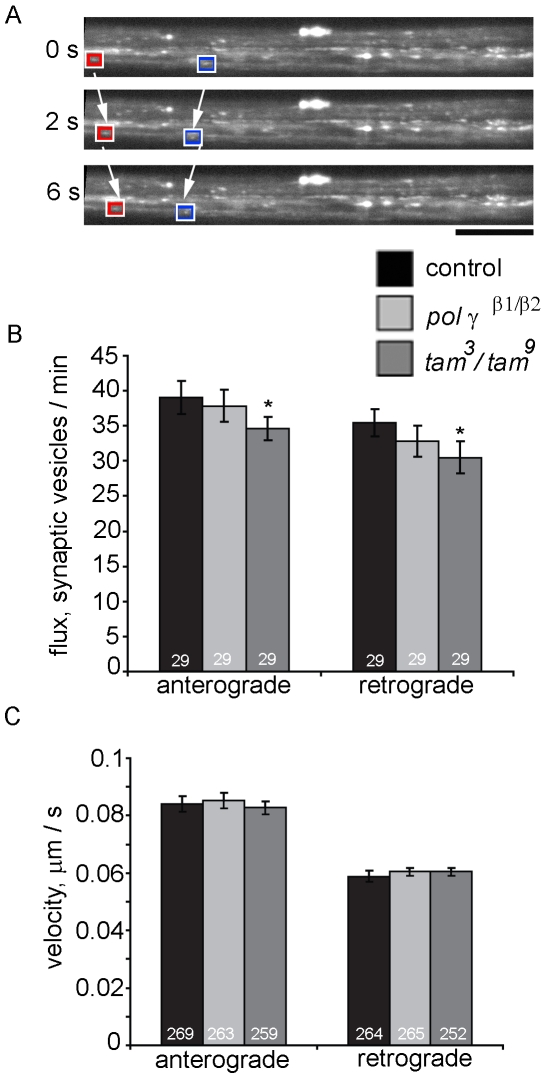
Flux of synaptic vesicle transport is moderately reduced in *tam* ^3^/*tam* ^9^ mutants whereas velocity remains unchanged. (A) *UAS-n-sybGFP* is expressed in segmental nerves of *Drosophila* larvae using the *D42*-GAL4 driver. Time lapse imaging of proximal region of medial nerves allows visualization of synaptic vesicle transport. Red box indicates anterograde while blue box indicates retrograde moving vesicle. (B) There is a moderate decrease in bidirectional synaptic vesicle flux in *tam* ^3^/*tam* ^9^ mutants while *pol γ ^β1/β2^* mutants show no significant difference in flux compared to wildtype controls. Numbers at the base of columns represent number of nerves assayed. (C) There is no significant change in average velocity of synaptic vesicle transport in either direction in the pol γ mutants. Numbers at the base of columns indicate number of synaptic vesicles traced. Error bars represent 95% confidence intervals. * indicates p<0.05 from Student's t-test.

To determine if the observed elevation of bidirectional flux and decrease in the velocity of kinesin mediated transport was global, we observed transport of synaptic vesicle precursors in *pol γ-^β1/β2^* and *tam* ^3^/*tam* ^9^ mutants (Videos S3 and S4). SNARE protein synaptobrevin (Vesicle Associated Membrane Protein) tagged to GFP was expressed in motor neurons using the *D42*-Gal4 driver, and its axonal transport was assayed in the segmental nerves using time lapse confocal imaging in crawling third instar *Drosophila* larvae (Figure 9A). Measurement of flux and velocity revealed that axonal transport of synaptic vesicle precursors was not altered severely by disruption of mtDNA replication. Although, *tam* ^3^/*tam* ^9^ mutants showed a slight decrease in flux, this transport behavior is in contrast to the elevation in bidirectional mitochondrial transport and could potentially be an indicator of depleted ATP levels. There was no significant difference in the velocity of anterograde and retrograde synaptic vesicle precursor transport in *pol γ-^β1/β2^*, *tam3*/*tam* ^9^ and control larvae (Figure 9B and C). The average flux and velocity of synaptic vesicle precursors with standard deviations are summarized in Table 1. We conclude that disruption of mitochondrial DNA replication does not cause global impairment of axonal transport.

## Discussion

Disruption of axonal transport and mitochondrial function are associated with many neurological diseases. While acute disruption of oxidative phosphorylation by mitochondrial poisons (CCCP in particular) can lead to a dramatic blockade of axonal transport [13], [19], there have been no studies that have examined the effect of mutations in genes that disrupt mitochondrial ATP synthesis on axonal transport *in vivo*. Here, we impaired genetically the mtDNA replication machinery to study axonal transport in *Drosophila*. Animal mtDNA encodes 13 essential polypeptides that constitute subunits of the oxidative phosphorylation complexes [41]. We began with the hypothesis that depletion of mtDNA would dramatically impair mitochondrial and synaptic vesicle precursor transport in axons. Surprisingly, we found that when mtDNA is depleted in pol γ mutants, bidirectional flux of mitochondrial transport is almost doubled, and there is a significant increase in mitochondrial density along the proximal axons. Furthermore, the flux of synaptic vesicle precursors is maintained at comparable levels suggesting that there is an organelle-specific regulation of transport in mtDNA depleted mutants.

The observed transport profiles of mitochondria and synaptic vesicle precursors suggest that normal oxidative phosphorylation is not critical for the sustenance of axonal transport. When mitochondrial ATP generation is disrupted, there is a consistent decrease in cellular ATP concentration: ∼30% reduction is observed in *Drosophila* [32], a ∼10–40% reduction in human cell lines [42], [43], and a ∼40–50% reduction in cerebellar granule cells [44]. Nonetheless, this is not as dramatic as would be expected based on the contribution that mitochondrial oxidative phosphorylation normally makes to ATP generation (*i.e.*, ∼90% of total ATP produced).

It is important to note that glycolysis is upregulated under oxidative stress and/or when oxidative phosphorylation is disrupted [42], [45]. For example, depletion of mitochondrial transcription factor B2 (*TFB2M*) of the mtDNA transcription machinery leads to a metabolic shift towards glycolysis in *Drosophila* that partially restores ATP levels [32]. Similarly, transcription factor A (*Tfam*) knockout mice display increased gene expression of several glycolytic enzymes [46]. Thus it is likely that glycolysis compensates for defective oxidative phosphorylation to sustain axonal transport in *pol γ-^β1/β2^* and *tam* ^3^/*tam* ^9^ mutants.

The observed increase in bidirectional mitochondrial flux was coupled with higher mitochondrial density in muscles and proximal nerves of *tam^3^/tam^9^* mutants. Higher density could be a consequence of two phenomena: increased mitochondrial fragmentation or addition of new mitochondria. To test these possibilities, we looked at electron micrographs of muscles and nerves from pol γ mutants. There was no sign of the morphological features (damaged or missing cristae) that typically accompany unusual mitochondrial fragmentation. Further, we did not see a significant difference in density of docked mitochondria in the nerves of *tam^3^/tam^9^* mutants. Together these data suggest that the observed increase in density and transport was due to addition of new mitochondria, albeit without mtDNA. This increase in mitochondrial number is consistent with the increased biogenesis observed in mitochondrial myopathies [46]–[48]. It would be logical for diseased cells to have a checkpoint to safeguard against proliferation of defective mitochondria. Yet our data, like those obtained previously in other systems [46]–[48], suggests that mitochondrial biogenesis increases when mitochondrial function is compromised.

A possible explanation for the increase in bidirectional mitochondrial transport is that it is a simple reflection of a greater mitochondrial density in the nerves of the mutant animals. In the control animals we found an anterograde flux of 10.15+/−2.48 mito/min (average+/−s.d.) and a density of 0.25+/−0.01 mito/µm^2^ (average+/−s.d.) in the proximal nerves. In the *tam^3^/tam^9^* animals we found an anterograde flux of 18.20+/−4.87 mito/min (average+/−s.d.) and a density of 0.28+/−0.01 mito/µm^2^ (average+/−s.d.). Whereas the increase in density was statistically significant in the *tam^3^/tam^9^* animals, it was small as compared to the increase in flux: the flux increased by 79% and the density increased by 12%. Furthermore, examination of the density of transported and docked mitochondria shows that there is little difference between the levels of docked mitochondria, but a significant increase in the number of transported mitochondria (Fig. 8B). Together these data argue strongly that bidirectional mitochondrial flux is increased when mtDNA replication is inhibited.

An additional factor that may influence mitochondrial flux is that mtDNA replication might require mitochondria to dock. If so, inhibition of mtDNA replication would naturally lead to elevated transport. This raises the question of “what percentage of mitochondria undergo mtDNA replication at a given time?” Based on EM analysis of mtDNA molecules in *Drosophila* [49], ∼1% of mtDNA molecules are replicating. Because mitochondria are typically thought to contain ∼10 copies of mtDNA, approximately 10% of all mitochondria might have replicating mtDNA. Whereas some mtDNA replication occurs along the axon, it appears that the majority occurs in the neuronal cell body [50], [51]. Thus, one could assume that along the axon somewhat less than 10% of all mitochondria have replicating mtDNA. Thus, whereas it is possible that a decrease in mtDNA replication could lead to an increase in mitochondrial axonal transport, it would be expected to represent only a modest increase.

We previously demonstrated using the dye JC1 that polarized mitochondria are transported in the anterograde direction and depolarized mitochondria are transported in the retrograde direction along the axon [13]. Our interpretation of this data was that following biogenesis newly synthesized mitochondria are transported out into the axon and damaged mitochondria are transported back to the cell body for degradation. While a recent study confirmed these results with JC-1, the investigators failed to see a correlation between membrane potential and direction of transport with the dye TMRM [52]. If we consider the results of the TMRM study at face value and assume that mitochondria, regardless of membrane potential, move randomly along the axon, the increased bidirectional transport of mitochondria could be interpreted simply as evidence that suggests an overall increase in mitochondrial trafficking when mtDNA replication is inhibited. Alternatively, a speculative interpretation based on the JC-1 data and our current results would suggest that inhibition of mtDNA replication increases bidirectional mitochondrial transport because biogenesis and degradation are both increased. In either case, elevated transport suggests an SOS response is occurring in a futile attempt to supply the axon with functional mitochondria.

Despite the increase in mitochondrial flux, we observed that velocity of kinesin mediated mitochondrial transport was reduced in pol γ mutants. Whereas flux reflects the demand and regulation of the cargo, transport velocity signifies motor activity. The precise reason for slower kinesin mediated mitochondrial transport is unclear. Kinesin velocity is known to be proportional to ATP concentration [53]. It is possible that kinesin-1, the main mitochondrial motor protein [14], is more sensitive to fluctuations in ATP concentration than cytoplasmic dynein. This could also account for a previous report that damaged mitochondria in cultured neurons from SOD1 mutant mice have decreased velocity of anterograde but not retrograde axonal transport [54]. In contrast, kinesin-3 is the major motor for synaptic vesicle transport [55], [56]. Kinesin-1 and kinesin-3 have dissimilar biophysical properties, adaptors and structure [57]. Conceivably, kinesin-3 may be less vulnerable to perturbations in ATP concentration than kinesin-1, explaining the normal velocity of synaptic vesicle precursor transport in *pol γ-^β1/β2^* and *tam* ^3^/*tam* ^9^ mutants. Although this impairment in anterograde mitochondrial transport is not severe by itself, it could contribute to peripheral neuropathy over a sustained time period as observed with some *POLG1* mutations [36], [37] and AZT treatment [58].

Mitophagy is an integral part of normal turnover and life cycle of mitochondria [59], [60]. In muscles of wildtype *Drosophila*, we noticed extra-nuclear dsDNA clusters localized to late endosomes/lysosomes, presumably from mitochondria that have been submitted for mitophagy. These clusters were conspicuous by their absence in *pol γ-^β1/β2^* and *tam* ^3^/*tam* ^9^ mutant muscles. The lack of any other known source of cellular dsDNA besides the nucleus and mitochondria suggests that they may be mtDNA aggregates from mitochondria that are destined for mitophagy. The absence of such lysosomal dsDNA clusters in *pol γ-^β1/β2^* and *tam* ^3^/*tam* ^9^ mutants clearly corroborates the severe depletion of mtDNA in these mutants. However, decreased mitophagy in *pol γ-^β1/β2^* and *tam* ^3^/*tam* ^9^ mutants remains an alternate explanation.

Interestingly, in all our studies we find that *tam* ^3^/*tam* ^9^ mutants have a more severe phenotype than *pol γ-^β1/β2^* mutants. In fact, even the heterozygous *tam* mutants displayed greater severity than the *pol γ−β* hetrozygotes. *tam (tamas)* encodes the catalytic subunit pol γ−α of the pol γ complex and is responsible for its DNA polymerase and exonuclease activities whereas *pol γ−β* encodes the accessory subunit pol γ−β, and enhances primer recognition and template-primer DNA binding [1]. A simple explanation for the difference in phenotypic severity could be that the mutations in the *tam* alleles are more disruptive than the specific mutations in the *pol γ−β* alleles. Alternatively, because the two subunits have clearly different functions, it is possible that deleterious mutations in the catalytic core have more serious consequences for the animal.

Defects of mtDNA are an important cause of neuropathy. Although much is known about the pathological ramifications of mtDNA defects in muscles, very little is understood about its cellular impact on neurons *in vivo* [61]. Based on our results, we postulate a speculative mechanistic model for the progressive neuropathy observed in mtDNA diseases: **1**) mutation or depletion of mtDNA leads to an increase in mitochondrial biogenesis to compensate for mitochondrial dysfunction; **2**) bidirectional mitochondrial transport is elevated in an attempt to supply the axon with mitochondria and as a consequence, the nerve is populated with dysfunctional mitochondria; **3**) these mitochondria consume cytosolic ATP to maintain their membrane potential [62] and potentially generate ROS, which increase cellular stress and lead to disrupted neuronal function and eventually cell death [63]; **4**) and in addition, subtle transport alterations such as reduction in kinesin-based mitochondrial transport and slight reduction in the flux of synaptic vesicles, contribute to distal neuropathy. Together, these cellular modifications could account for the sustained progression of neuronal pathology in mtDNA diseases. This model raises the intriguing question of whether cellular stress would be decreased in mtDNA diseases if mitochondrial biogenesis was intentionally inhibited, or if mitochondria lacking mtDNA have an undiscovered neuroprotective role.

This is the first study to monitor directly mitochondrial trafficking *in vivo* when mtDNA replication is genetically disrupted. While future studies are warranted to document conclusively the relevance of this work to mtDNA disease, our current findings provide potentially important and counter-intuitive insights into the biology of mitochondria in neurons.

## Materials and Methods

### Drosophila Stocks and Culture

Standard cornmeal fly medium was used and all stocks maintained at 25°C. *UAS-n-Syb-GFP* flies were obtained from Dr. Mani Ramaswami, University of Arizona, Tucson and *UAS-mtGFP* line from Dr. William Saxton, University of California, Santa Cruz. All other stocks were obtained from the Bloomington Stock Center, Indiana. Canton-S flies were used as the wildtype control strain unless otherwise mentioned. *UAS-mtGFP* was expressed in segmental nerves using the *D42*-Gal4 driver [14]. The *D42* driver predominantly expresses in motor neurons along with a few body wall sensory neurons, and the salivary glands in the larva [33]. Mutations in the 34D locus on the second chromosome of *Drosophila* cause a disruption of the 125-kDa catalytic subunit (pol γ−α) of mitochondrial pol γ [27], encoded by the gene *tamas*. For our analysis, we used two hypomorphic alleles of this gene, *tam^3^* and *tam^9^* that have a glutamate to alanine conversion at nucleotide position 1783, and a 5-bp deletion beginning at nucleotide position 3371, respectively. The gene encoding the accessory subunit of pol γ in *Drosophila* (pol γ−β) is *pol γ−β*, located in the 34D subdivision of the left arm of the second chromosome, 3.8 kb distal from the gene encoding the catalytic subunit. The mutant allele *pol γ-β^1^* is an EMS-induced mutation resulting in a glutamate substitution of a highly conserved glycine residue in the N-terminal domain of the accessory subunit. The *pol γ-β^2^* allele is a spontaneous mutation caused by an in-frame 74-bp insertion in the N-terminal domain that creates a premature stop [9]. Both pol γ mutants are homozygous lethal at the late larval third instar stage. To avoid phenotypic expression of any unknown background mutations, we used heteroallelic mutant combinations of *tam^3^/tam^9^* for pol γ−α studies and *pol γ-β^1^/pol γ-β^2^* for pol γ−β.

### Immunohistochemistry and PicoGreen Staining

Larvae were dissected in Ca^2+^ free saline and fixed for 20 min in 4% paraformaldehyde. Next, they were rinsed in phosphate-buffered saline (PBS) with 0.1% Tween-20 and after 30 min of blocking in 10% normal goat serum, incubated overnight at 4°C in primary antibody solution, followed by washes in PBST with three changes, and further incubation in PicoGreen dye and fluorescently-conjugated secondary antibody solution in PBST for 2 h at room temperature. Then the samples were washed in PBS and mounted under a cover glass. We used mouse anti-mitochondrial complex V monoclonal antibody at 1∶500 (MitoSciences, Eugene, OR, USA), guinea pig anti-spin at 1∶250 (Graeme Davis, University of California, San Francisco) and PicoGreen dye (Invitrogen, USA) at 1∶200. The following fluorescently labeled secondary antibodies were used: goat anti-mouse Alexa 568 (Invitrogen, USA) and goat anti-guinea pig Alexa 568 (Molecular Probes, USA).

### Mitochondrial and mtDNA Nucleoid Density

For measuring density, 3D reconstruction images of segmental nerves and muscle fibres 6/7 in abdominal segments A3–A6 in third instar *Drosophila* larvae were used. For muscles, a 55 µm×55 µm single frame stained with anti-complex V antibody or PicoGreen was divided into a grid of 64 regions of interest (ROI). A random list of numbers (1–64) was generated on Microsoft Excel and the number of mitochondria/mtDNA nucleoids was counted in eight of the corresponding ROI number for each frame and averaged over the area to yield density/µm^2^. (Figure 2B). For segmental nerves, *UAS-mtGFP* driven by *D42*-Gal4 was imaged in the proximal, medial and distal regions of the segmental nerves; the regions were divided into 100 µm^2^ bins and total mitochondria counted and averaged over that area to yield mitochondria/µm^2^ (Figure 4A and B).

### Southern Blotting

Total DNA was purified from third instar larvae by standard methods. DNA (3 µg) was cleaved with *Xho*I, which cuts mtDNA once, fractionated in a 0.8% agarose gel/TBE and transferred to a nylon membrane (Amersham Pharmacia Biotech). Hybridization was carried out for 16 h at 65°C in 10 mM sodium phosphate pH 7.4/0.5% SDS. Filters were washed three times for 10 min at room temperature with 4× SSC containing 0.1% SDS, once for 30 min at 65°C with 0.1× SSC containing 0.1% SDS. Blots were probed with radiolabeled DNAs for the mitochondrial gene ATPase 6 and the nuclear histone gene cluster.

### Electron Microscopy

The nerves and muscles of four different strains of *Drosophila* were examined: *pol γ-^β1/β2^* (n = 3), *tam* ^3^/*tam* ^9^ (n = 3), Canton-S (n = 2), and animals with *UAS-mtGFP; D42*-Gal4 in wildtype background (n = 2). Third instar larvae, 90–100 hrs after egg laying, were dissected in cold, Ca^2+^ free saline (125 mM NaCl, 4 mM MgCl_2_, 10 mM NaHCO_3_, 2 mM NaH_2_PO_4_, 5 mM trehalose, 40 mM sucrose, 10 mM Hepes, pH 7.2). After pinning in Sylgard dishes, they were rinsed in fresh, cold, Ca^2+^ free saline and then fixed for one hour with cold fixative (4% paraformaldehyde w/v and 1% glutaraldehyde v/v in Millonig's phosphate buffer, ph 7.2). They were then unpinned and fixed for an additional hour in fresh fixative in scintillation vials. After 12 hours rinse with 5 changes, in 0.1 M phosphate buffer, they were postfixed for two hours with 1% OsO_4_ in 0.1 M phosphate buffer. Following an overnight rinse in 0.1 M phosphate buffer, samples were dehydrated through a graded series of ethanol solutions, then propylene oxide. They were infiltrated overnight in 50% Poly/Bed 812 Araldite resin in proplylene oxide, and then 8 hours in 100% Poly/Bed 812. After placing in flat embedding molds, the resin was hardened for two days at 60°C. Animals were sectioned perpendicular to the long axis and thick sections photographed in order to map and identify the individual muscles. Thin sections were photographed with a JEOL 100CX electron microscope at 60 kV. Negatives were digitized at 600, 1200, 2400 or 3200 pixels per inch with an Epson V750 Pro flat bed scanner equipped with a negative carrier.

### Image Acquisition and Analysis of Axonal Transport

Crawling third instar *Drosophila* larvae were selected and anaesthetized in halocarbon oil 700 (Sigma) with 10–25% chloroform, titrated to levels just sufficient to inhibit significant muscular contraction. The larvae were then mounted between a slide and coverslip and were imaged for no more than 15 minutes at ∼25°C (Figure 7A). All images were acquired on a swept field confocal microscope with NIS software using a Nikon TE2000-E inverted microscope and a PlanApo 60X oil objective, NA 1.4. The aperture and exposure were set at 70-slit and 100 ms, respectively, and images were captured at 2 s intervals for total time of 7 min for a time-lapse series.

NIS files were opened in ImageJ, and frames were aligned using the StackReg plugin with rigid body settings. The two medial nerves at the base of the ventral nerve cord were selected for each analysis (Figure 7B), cropped and rotated, using TJ Rotate with cubic-B-spline interpolation, so that the axons were always oriented horizontally with the cell body on one side and the synapse on the other. These images were re-sliced and z-projected using the sum-slices option to generate kymographs. The kymographs were opened in Adobe Photoshop, image color depth was converted from 16 bits/pixel to 8 bits/pixel and color inverted to facilitate better visibility of transport events.

For the flux, total numbers of transport events in each direction were counted at three different positions along each axon at different time points and an average was taken. This was considered one data point of transport in units of mitochondria/min or synaptic vesicles/min. For velocity of transport, lines were hand-traced over the path of mitochondria or synaptic vesicle precursors on the kymographs, on different layers in Adobe Photoshop and the slope calculated for each (Figure 7C). These results were then exported to Microsoft Excel to calculate average velocity of transport in units of µm/s.

For measurement of docked mitochondria, total numbers of mitochondria that remained stationary during the entire duration of observation were counted and divided by the length of nerve imaged to yield docked mitochondria/µm. Data from 10–12 nerves was averaged in each case. These observations were made in the same regions in which flux was measured.

## Supporting Information

Video S1 Axonal transport of mitochondria in *UAS-mitoGFP, D42-*Gal4 *Drosophila* larval controls. Crawling third-instar *Drosophila* larvae expressing mitoGFP were anesthetized with chloroform and imaged intact on a swept-field confocal microscope. Observations were made every 2 s on a single plane in a temperature controlled chamber set at 25°C. Transport was observed in the most medial segmental nerves close to the ventral nerve cord. This video is accelerated to 15X of normal speed.
(2.59 MB MOV)

Video S2 Axonal transport of mitochondria in pol γ-α mutants (*tam^3^/tam^9^*). Bidirectional mitochondrial flux is elevated in *tam^3^/tam^9^* mutants, but velocity of kinesin mediated anterograde transport is slower than control animals. Crawling third-instar larvae expressing mitoGFP were anesthetized with chloroform and imaged intact on a swept-field confocal microscope. Observations were made every 2 s on a single plane in a temperature controlled chamber set at 25°C. Transport was observed in the most medial segmental nerves close to the ventral nerve cord. This video is accelerated to 15X of normal speed.
(1.90 MB MOV)

Video S3 Axonal transport of synaptic vesicle precursors in *UAS-n-sybGFP, D42-*Gal4 *Drosophila* larval controls. Crawling third-instar *Drosophila* larvae expressing synaptobrevin-gfp (n-syb-GFP) were anesthetized with chloroform and imaged intact on a swept-field confocal microscope. Observations were made every 2 s on a single plane at a temperature of 25°C. Transport was observed in the most medial peripheral nerves close to the ventral nerve cord. This video is accelerated to 15X of normal speed.
(6.34 MB MOV)

Video S4 Axonal transport of synaptic vesicle precursors in pol γ-α mutants (*tam^3^/tam^9^*). No striking differences were seen in transport of syb-GFP tagged vesicles between the control and mutant animals. Bidirectional flux of *tam^3^/tam^9^* mutants is slightly reduced while velocity remains unchanged. Crawling third-instar larvae expressing synaptobrevin-GFP (syb-GFP) were anesthetized with chloroform and imaged intact on a swept-field confocal microscope. Observations were made every 2 s on a single plane at a temperature of 25°C. Transport was observed in the most medial peripheral nerves close to the ventral nerve cord. This video is accelerated to 15X of normal speed.
(6.57 MB MOV)
